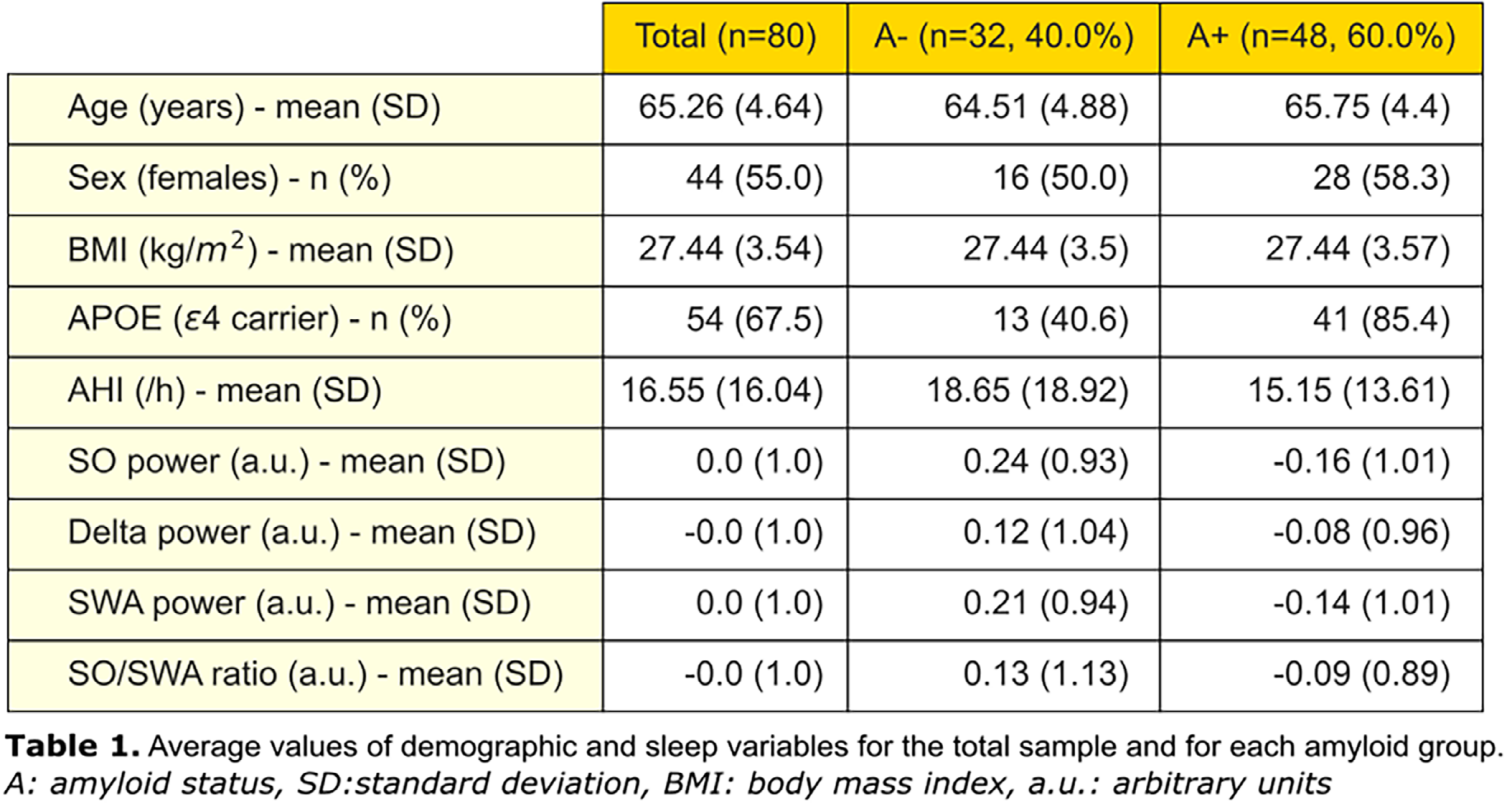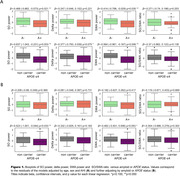# Early amyloid accumulation and APOE‐ε4 are associated with reduced non‐rapid eye movement slow‐wave activity in cognitively unimpaired adults

**DOI:** 10.1002/alz.086410

**Published:** 2025-01-09

**Authors:** Núria Tort‐Colet, Ana Fernández‐Arcos, Laura Stankeviciute, Carolina Minguillon, Laura Hernández, Iva Knezevic, Gonzalo Sánchez‐Benavides, Marc Suarez‐Calvet, Sebastian C Holst, Pilar Garces, Thomas Mueggler, Henrik Zetterberg, Kaj Blennow, Amaia Muñoz‐Lopetegui, Aurora Arqueros, Álex Iranzo, Juan Domingo Gispert, Jose Luis Molinuevo, Oriol Grau‐Rivera

**Affiliations:** ^1^ Barcelonaβeta Brain Research Center (BBRC), Pasqual Maragall Foundation, Barcelona Spain; ^2^ Hospital del Mar Research Institute, Barcelona Spain; ^3^ Barcelonaβeta Brain Research Center (BBRC), Barcelona Spain; ^4^ Centro de Investigación Biomédica en Red de Fragilidad y Envejecimiento Saludable (CIBERFES), Instituto de Salud Carlos III, Madrid Spain; ^5^ Centro de Investigación Biomédica en Red de Fragilidad y Envejecimiento Saludable (CIBERFES), Madrid Spain; ^6^ Hospital del Mar Research Institute (IMIM), Barcelona Spain; ^7^ Servei de Neurologia, Hospital del Mar, Barcelona Spain; ^8^ Roche Pharma Research and Early Development, Neuroscience and Rare Diseases, Roche Innovation Center Basel, F. Hoffmann ‐ La Roche Ltd, Basel Switzerland; ^9^ Neuroscience and Rare Diseases, Roche Pharma Early Research and Development, F. Hoffmann‐La Roche Ltd, Basel Switzerland; ^10^ Hong Kong Center for Neurodegenerative Diseases, Hong Kong China; ^11^ Department of Neurodegenerative Disease, UCL Queen Square Institute of Neurology, University College London, London, ‐ United Kingdom; ^12^ UK Dementia Research Institute at UCL, London United Kingdom; ^13^ Department of Psychiatry and Neurochemistry, Institute of Neuroscience and Physiology, the Sahlgrenska Academy at the University of Gothenburg, Mölndal Sweden; ^14^ Department of Psychiatry and Neurochemistry, Institute of Neuroscience and Physiology, The Sahlgrenska Academy at the University of Gothenburg, Mölndal Sweden; ^15^ Clinical Neurochemistry Laboratory Sahlgrenska University Hospital, Mölndal Sweden; ^16^ Department of Psychiatry and Neurochemistry, Institute of Neuroscience and Physiology, University of Gothenburg, Mölndal Sweden; ^17^ Neurology Service, Hospital Clínic de Barcelona and Institut D'Investigacions Biomèdiques August Pi i Sunyer (IDIBAPS), Barcelona Spain; ^18^ Centro de Investigación Biomédica en Red sobre Enfermedades Neurodegenerativas (CIBERNED), Barcelona Spain; ^19^ Hospital del Mar Research Institute, Barcelona, Barcelona Spain; ^20^ Centro de Investigación Biomédica en Red Bioingeniería, Biomateriales y Nanomedicina (CIBER‐BBN), Instituto de Salud Carlos III, Madrid Spain; ^21^ H. Lundbeck A/S, Copenhagen Denmark

## Abstract

**Background:**

Poor sleep is associated with cognitive decline, and ∼45% of Alzheimer’s disease (AD) patients experience sleep disturbances. Emerging evidence suggests that reduced non‐rapid eye movement (NREM) slow wave sleep (SWS) is linked to amyloid accumulation and APOE ε4‐related genetic vulnerability to AD. Here, we investigate the effects of amyloid and *APOE* status on SWS, and their interaction, in a cohort of cognitively unimpaired (CU) individuals at higher risk of AD.

**Method:**

We analyzed data from 80 sleep medication free participants (65.26 years old on average, 55% females, Table 1) from the AlfaSleep project, which involves characterization with polysomnography, *APOE* genotyping, CSF amyloid‐β (Aβ) 40 and Aβ42 (Roche NeuroToolKit), and [^18^F]flutemetamol‐PET, among other measures. Participants were classified as amyloid positive (A+) using either CSF Aβ 42/40 ratio (<0.071, N = 75), or amyloid PET (>12 Centiloids, N = 5) thresholds for early amyloid deposition.

We computed average power spectral densities (PSD) from NREM sleep 30‐s epochs in frontal electroencephalogram channels, and power of slow oscillations (SO), delta and slow wave activity (SWA) as the area under the PSD curve between 0.5‐1Hz, 1‐4Hz and 0.5‐4Hz, respectively. We log‐transformed SWS metrics due to non‐normal distribution. We used linear regression models to test the main effect of amyloid or *APOE* status on SWS metrics in separated models, and next we entered both in the same model. Finally, we the tested interaction between *APOE* and amyloid status on SWS metrics. Models were adjusted by age, sex, and apnea‐hypopnea index (AHI).

**Result:**

Both A+ status and *APOE*‐ε4 carriership were associated with significantly lower power in SO and SWA in separated models (Figure 1A). When combining both in the same model, only the effect of *APOE* on SO power remained significant, as well as a trend for lower SWA (Figure 1B). Interaction analyses yielded no significance.

**Conclusion:**

Early amyloid accumulation and *APOE*‐ε4 carriership are associated with SWS disruption in CU adults, with *APOE*‐ε4 carriers probably being particularly vulnerable to SWS disruption. Longitudinal studies spanning younger age individuals are needed to characterize the contribution of *APOE*‐ε4 to SWS disruption and to test whether preventive interventions targeting SWS could help to reduce AD dementia risk.